# Metoclopramide-induced central nervous system depression in the chicken

**DOI:** 10.1186/1746-6148-1-6

**Published:** 2005-10-14

**Authors:** Muna HI Al-Zubaidy, Fouad K Mohammad

**Affiliations:** 1 Department of Physiology, Biochemistry and Pharmacology, College of Veterinary Medicine, University of Mosul, PO Box 11136, Mosul, Iraq

## Abstract

**Background:**

Metoclopramide is a dopamine D2-receptor antagonist used as an antiemetic and gastroprokinetic agent in man and animals. The drug causes sedation as a side effect in man. Such a sedative action of metoclopramide has not been documented in the chicken as the drug is not used clinically in this species. The present study examines the central nervous system depressant effects of metoclopramide in 7–14 days old broiler chicks.

**Results:**

Injection of metoclopramide at 50, 100 and 200 mg/kg, subcutaneously (s.c.) induced sedation in the chicks in a dose dependent manner. The chicks manifested, within 3.6–19 minutes of metoclopramide injection, signs of sedation characterized by drooping of the head and wings, closed eyelids, reduced motility and decreased distress calls. The duration of sedation ranged between 37.2 to 163.4 minutes. Metoclopramide at 100 and 200 mg/kg induced, within 12.2 and 6.2 minutes, sleep (loss of righting reflex) for 43.8 and 158.6 minutes, respectively. The median effective doses of metoclopramide for induction of sedation and sleep in the chicks were 11 and 53 mg/kg, s.c., respectively. Lower doses of metoclopramide (5 and 10 mg/kg, s.c.) significantly decreased the open-field activity of the chicks and increased the durations of their tonic immobility. All treated-chicks recovered from the central nervous system depressant effect of metoclopramide without any observable adverse effects.

**Conclusion:**

The data suggest that metoclopramide induces central nervous system depression in chicks, and the drug could have potential clinical applications as a sedative-hypnotic agent in avian species not intended for human consumptions.

## Background

Metoclopramide (methoxychloroprocainamide) is a dopamine D2-receptor antagonist used as an antiemetic and gastroprokinetic agent in man [1] as well as in dogs [2-4] and cats [3-5]. The drug also has serotonergic effects [6] and indirect parasympathomimetic activity [7,8]. Metoclopramide has been used experimentally in pigeons as an antiemetic agent at 10, 20 and 40 mg/kg, body weight [9]. Antidopaminergic drugs such as phenothiazine tranquilizers are known to induce a state of sedation in different animal species, including the chicken [3,4,10]. Metoclopramide treatment has been reported to cause sedation in man [1,11,12]. Such a sedative action of metoclopramide, though a side effect, has not been documented in the chicken as the drug is not used clinically in this species. In this communication, we present the central nervous system (CNS) depressant activity of metoclopramide in 7–14 days old broiler chicks. This age group of the chicken is suitable for examining sedative effects of CNS depressants [13-15].

## Results

Injection of metoclopramide at 50, 100 and 200 mg/kg, subcutaneously (s.c.) induced sedation in the chicks in a dose dependent manner. The chicks manifested within 3.6–19 minutes of metoclopramide injection (Table 1) signs of sedation characterized by drooping of the head and wings, closed eyelids, reduced motility and decreased distress calls. The duration of sedation ranged between 37.2 to 163.4 minutes and it was also dose dependent (Table 1). Metoclopramide at 100 and 200 mg/kg induced, within 12.2 and 6.2 minutes, sleep (loss of righting reflex) in the chicks for 43.8 and 158.6 minutes, respectively (Table 1).

**Table 1:** Metoclopramide -induced sedation and sleep (loss of righting reflex) in chicks

Metoclopramide (mg/kg, subcutaneously)	Latency to onset of sedation (minute)	Latency to onset of sleep (minute)	Duration of sedation (minute)	Duration of sleep (minute)	Recovery time (minute)
50	19.0 ± 2.2	none	37.2 ± 7.6	none	43.4 ± 7.8
100	8.8 ± 2.5*	12.2 ± 2.2	66.4 ± 5.5*	43.8 ± 3.5	74.6 ± 6.4*
200	3.6 ± 0.5*	6.2 ± 0.5†	163.4 ± 17.4*†	158.6 ± 23.3*†	168.4 ± 16.6*†

Values are mean ± SE of 5 chicks/group.

* Significantly different from the 50 mg/kg treatment group, P < 0.05.

† Significantly different from the 100 mg/kg treatment group, P < 0.05.

A control group of chicks was also treated with physiological saline solution (5 ml/kg, subcutaneously).

The median effective doses (ED50s) of metoclopramide for the induction of sedation and sleep in the chicks, as determined by the up-and-down method, were 11 and 53 mg/kg, s.c., respectively (Table 2). Metoclopramide at lower doses (5 and 10 mg/kg, s.c.) also caused CNS depression in chicks; the drug significantly decreased the open field activity of the chicks and significantly increased the durations of their tonic immobility (Table 3). All chicks recovered smoothly from metoclopramide-induced CNS depression and none of them suffered from adverse effects or died during the study.

**Table 2:** Determination of median effective doses (ED50) of metoclopramide for induction of sedation and sleep (loss of righting reflex) in chicks by the up-and-down method*

Variable	Result
Sedation	
ED50	11 mg/kg, subcutaneously (s.c.)
Range of the doses used	25-5 = 20 mg/kg, s.c.
Initial dose	25 mg/kg, s.c.
Last dose	10 mg/kg, s.c.
Number of chicks used	8 (XXXXOOXO)
Increase or decrease in the dose	5 mg/kg, s.c.
Range of latency to induce sedation	20-5 = 15 minutes
Range of duration of sedation	44-7 = 37 minutes
Sleep	
ED50	53 mg/kg, s.c.
Range of the doses used	100-50 = 50 mg/kg, s.c.
Initial dose	100 mg/kg, s.c.
Last dose	60 mg/kg, s.c.
Number of chicks used	9 (XXXXXOXOX)
Increase or decrease in the dose	10 mg/kg, s.c.
Range of latency to induce sleep	12-2 = 10 minutes
Range of duration of sleep	67-11 = 56 minutes

*Dixon [18].

X = sedation or sleep; O = no sedation or sleep

**Table 3:** Effect of metoclopramide on open-field activity and tonic immobility test in chicks

Metoclopramide (mg/kg, subcutaneously)	Squares crossed/5 minutes	Duration of immobility (second)
0 (saline-control)	3.5 ± 1.4	10.5 ± 2.7
5	1.0 ± 1.0*	54.9 ± 18.1
10	0.1 ± 0.1*	130.6 ± 26.3*†

Open-field activity was measured 20 min after drug administration. Tonic immobility test was done immediately after the open-field test.

Values are mean ± SE of 8 chicks/group.

* Significantly different from the respective control values, P < 0.05.

† Significantly different from the 5 mg/kg treatment group, P < 0.05.

## Discussion

Sedation has been reported clinically in man treated with regular therapeutic (or higher) doses of metoclopramide [1,11,12]. This effect has not been quantitatively reported in animals, especially in avian species. In the present report, metoclopramide -induced CNS depression in the chicks was concluded depending on the signs of sedation and sleep described in avian species [13-15]. The durations of sedation and sleep were also quantitatively reported (Table 1). The ED50s of metoclopramide for the induction of sedation and sleep were calculated for the first time in chicks by the up-and down method. Open-field activity and tonic immobility tests presented additional evidences for the CNS depressant activity of metoclopramide in the chicks. Little information are available on the pharmacological profile of metoclopramide in birds. The drug affects gastrointestinal motility in hispaniolan parrots [16] and prevents reserpine-induced emesis in pigeons [9]. Further, metoclopramide was found to increase gastrointestinal tract motility and inhibit plasma cholinesterase activity in chicks [17]. These effects are similar to those found in other animal species [2-5,8] and further suggest that the sedative activity of metoclopramide in the chicken should be potentially expected.

Metoclopramide depression is usually considered a side effect in man [1,11,12] and possibly in animals. Sedation could then be an additional pharmacological (side) effect of this drug to be expected in avian species. Further exploration of the potential research and possibly therapeutic applications of this drug is needed as a sedative agent in avian species not intended for human consumption, as the drug is not approved for use in food producing animals. The chicks in the present study were successfully used as a model to show the CNS depressant action of metoclopramide. Chicks were reported to be a suitable animal model for examining the CNS depressant action of drugs [13-15]. The sedative-hypnotic doses (50, 100 and 200 mg/kg, s.c.) of metoclopramide used in the present study are higher than the therapeutic ones used in dogs and cats [2-5]. However, the doses (especially the ED50s) of metoclopramide used in the present study are close to its antiemetic ones (10, 20 and 40 mg/kg, body weight, orally) used in pigeons treated with reserpine [9]. Using the open-field activity test, metoclopramide-induced CNS depression could be detected in the present study even at a dose as low as 5 mg/kg, s.c. (Table 3). Further, species differences in the magnitude of response to metoclopramide doses or its quality should be expected between mammals and birds or even among various avian species. Such a species difference has been reported with sedatives like xylazine [4,10,15].

Overall, the findings of the present study indicate the sedative and hypnotic (CNS depressant) effects of metoclopramide in chicks. These effects could be attributed to the antidopaminergic action of metoclopramide at the level of the CNS [1]. Centrally acting antidopaminergic drugs such as phenothiazine derivatives and butyrophenones are known to induce CNS depression in different animals as well as in avian species [3,4,10].

## Conclusion

The data suggest that metoclopramide induces CNS depression in chicks, and the drug could have potential research and clinical applications as a sedative-hypnotic agent in avian species not intended for human consumption.

## Methods

Unsexed, 7–14 days old, broiler chicks (body weight 52–95 g) were used. They were maintained in batches of 20–30 chicks at a time in a room at a temperature of 30–34°C-controlled by electric heaters with constant lighting. Litter consisted of wood shavings. Water and feed were given ad libitum. The chicks were treated s.c. with physiological saline solution at 5 ml/kg body weight (control group) or with metoclopramide HCl (Yuhan Corp., South Korea) at 50, 100 and 200 mg/kg body weight. Metoclopramide was dissolved in physiological saline solution, and the volume of administration was at 5 ml/kg body weight. The site of s.c. injection was on either lateral side of the chest. Care was taken so that leakage did not occur during or after the drug administration from the site of injection. The choice of metoclopramide doses was based on preliminary experiments in chicks in which doses more than 20 mg/kg, s.c. induced signs of sedation characterized by drooping of the head, closed eyelids, reduced motility or immotility, and decreased distress calls as well as recumbeny. After the injection of metoclopramide the chicks were monitored for the onset of sedation (drooping of the head) and sleep (loss of righting reflex after placing the chick on one side). The durations of sedation and sleep as well as recovery times were recorded too. Recovery time was the time from the onset of sedation until the chick moved freely. The ED50s of metoclopramide for the induction of sedation or sleep in the chicks were determined by the up-and-down method [18].

Further, the CNS depressant action of metoclopramide at lower doses (5 and 10 mg/kg) was also monitored by examining the open field activity [13,19] of the chicks and then subjecting them to the tonic immobility test [20]. In this experiment the chicks were treated s.c. with either physiological saline solution (control) at 5 ml/kg, or with metoclopramide at 5 and 10 mg/5 ml saline/kg body weight. Twenty minutes after the injection, each chick was placed alone on the center of the arena of an open field box (90 × 60 × 50 cm); the arena was divided into 24 equal squares and 50 g of wheat grains were scattered on the surface [13]. Open field activity was monitored by counting, within 5 minutes, the number of squares entered by both feet. After the open field activity test, each chick was subjected to tonic immobility test [20] by holding the chick in both hands and placing it on a wooden table for 15 seconds, then the hands were withdrawn and the chick was timed to upright itself and standing.

All experiments complied with regulations addressing animal use, and proper attention has been given to ethical consideration towards the chicks used in the present study. The data were statistically analyzed by one way analysis of variance followed by the least significant difference test [21]. Non-parametric data (open-field activity) were subjected to Mann-Whitney-U-test [22]. The level of significance was at P < 0.05.

## Authors' contributions

MHIA executed the experiments, shared in statistical analysis and shared in drafting the manuscript.

FKM conceptualized the study, designed the experiments, supervised drug administration and behavioral tests, shared in statistical analysis and drafted the manuscript.

The authors read and approved the manuscript.
